# Reading Music or Reading Notes? Rethinking Musical Stimuli in Eye-Movement Research

**DOI:** 10.3390/jemr19010003

**Published:** 2025-12-29

**Authors:** Katarzyna Julia Leikvoll

**Affiliations:** Department of Music, University of Bergen, 5007 Bergen, Norway; julia.leikvoll@uib.no

**Keywords:** eye movements, music reading, musical stimuli, musical syntax

## Abstract

This article examines the nature of musical stimuli used in eye-movement research on music reading, with a focus on syntactic elements essential for fluent reading: melody, rhythm, and harmony. Drawing parallels between language and music as syntactic systems, the study critiques the widespread use of stimuli that lack coherent musical structure, such as random pitch sequences or rhythmically ambiguous patterns. Eight peer-reviewed studies were analyzed based on their use of stimuli specifically composed for research purposes. The findings reveal that most stimuli do not reflect authentic musical syntax, limiting the validity of conclusions about music reading processes. The article also explores how researchers interpret the concept of “complexity” in musical stimuli, noting inconsistencies and a lack of standardized criteria. Additionally, it highlights the importance of considering motor planning and instrument-specific challenges, which are often overlooked in experimental design. The study calls for more deliberate and informed stimulus design in future research, emphasizing the need for syntactically meaningful musical excerpts and standardized definitions of complexity. Such improvements are essential for advancing the understanding of syntactic processing in music reading and ensuring methodological consistency across studies.

## 1. Introduction

Reading is a necessary skill for anyone who wants to be part of today’s society. The reading process has therefore been of great interest to researchers for several decades [1]. New techniques such as examining eye movements and activity in different parts of the brain have provided opportunities for a relatively accurate description of the reading process. Research into music reading seen in this context has been given considerably less priority [2,3,4,5].

Both language and music are syntactic systems governed by the hierarchical organization of elements into meaningful structures [6,7]. Experiments investigating eye movements during reading often focus on understanding how comprehension is affected when various elements of the text are altered or manipulated [8]. Each word in a text serves a relatively unambiguous function that can be identified and described. Grammatical and orthographic rules guide the construction of letters into words, words into sentences, and sentences into longer paragraphs. As a result, language texts are relatively easy to process.

Similarly, the notes in a music score are organized into meaningful units that serve different functions within the read or performed musical text. However, these units are not as clearly visually defined as words and sentences. Despite this, researchers frequently adopt experimental designs from language reading studies to investigate music reading. For decades, experiments examining eye movements have used language-based paradigms, substituting linguistic stimuli with musical notation [4,5,9,10].

Most eye-movement research in reading focuses on meaningful units composed of letters, namely, words and word groups, their lexical processing, and the reader’s perceptual span [1,11]. Numerous studies over the past decades have shown that skilled readers fixate on nearly every word in a text, and that the ability to extract visual information quickly and efficiently is fundamental to proficient reading [12]. Eye movements during reading are largely, if not entirely, driven by lexical linguistic processing [13]. Useful graphemic information is typically extracted from the first three letters of the word to the right of the fixation point [1]. Overall, the word appears to be the primary unit of reference in eye-movement analysis within reading research [12].

Music is perceived as sounds organized in relation to one another, rather than as a stream of isolated elements. One of the fundamental aspects of this organization is the similarity between different elements, which may manifest through repetition of phrases, rhythms, or harmonic patterns. Consequently, the ability to quickly organize written musical symbols into larger, meaningful patterns, and to retrieve them automatically, is essential for fluent music reading [14,15,16,17]. This ability to segment written music stems from a substantial inventory of meaningful units stored in the musician’s long-term memory.

A music score comprises meaningful patterns, such as rhythmic, melodic, harmonic, or timbral structures (depending on the repertoire) which fluent music readers automatically chunk into larger units of information [2,15]. In terms of visual processing, these units can be considered analogous to words in a language text, providing both understanding and the ability to anticipate what comes next. However, this aspect of musical stimuli seems to not have been sufficiently considered in research design. Musical stimuli in experimental studies often consist of isolated or random pitches and note values that cannot be chunked into meaningful units [18,19,20,21]. While such designs may offer insights into low-level perceptual processes, especially when memory-based templates are unavailable, they lack the syntactic coherence of real music. As with language studies using shuffled text [22], these stimuli can reveal how processing becomes less efficient without syntactic structure, but they do not reflect fluent reading. Although early studies like Waters [20,23] used limited stimuli and outdated technology, the more pressing issue is how similar limitations persist in recent research, where findings from artificial stimuli are sometimes generalized to real-world music reading.

The research question explored in this article is: *How can the nature of a music score as a visual stimulus influence the interpretation of music reading research results?* The article focuses on the reading process using the alphabetical notation system in language, the Western staff notation system, and the reading of Western tonal music. Western music notation, as a globally recognized system, transcends language barriers. Moreover, the ability to read music is valuable for professionals, amateurs, and their instructors alike. Therefore, eye-movement research in music holds broad potential for both theoretical insight and practical application [5].

## 2. Text and Music Reading

Language reading and music reading as cognitive processes have much in common and have frequently been compared to each other [2,7,24,25,26,27,28,29]. People process linguistic and musical stimuli as syntactic structures [7]. It is much easier to remember sequences that match the grammatical rules than those that do not, regardless of whether the sequence is meaningful at the semantic level (e.g., “The blue cat quickly ran above the table” compared to “Quickly table ran cat blue the above”) [7] (p. 241). In music, it is easier to remember sequences that apply the conventional rules of tonality and typical harmonic relations. This effect increases with the amount of knowledge about the music idiom that is being performed [28].

The basic level of the musical structure is related to scale and its pitches. All tones in a piece based on Western harmony are perceived in relation to the seven notes known as scale degrees, with a stable tonal center [7]. The durations of events are typically structured in simple integer ratios. Most often, each event lasts one, two, three, or four times the length of the shortest event [30]. They create “horizontal” organization of music. Musical syntax allows for the simultaneous use of multiple tones to form chords and create harmony. Chord syntax refers to the “vertical” organization of tones in music. A key element of musical syntax is the tension-relaxation relationship between chords, which is crucial in the structure of a musical piece. Harmonic variation involves contrasts between tension and relaxation. Like language syntax, the order of elements in harmonic structures is essential [6,7]. Scales and their chords are fundamental components of a key. The selection of keys used in a piece of music (such as in modulation) is always deliberate.

In language, syntax helps express the meaning of “who did what to whom,” essentially forming the conceptual structure of reference and predication in sentences. Similarly, in music, syntax supports the meaning through the pattern of tension and resolution that both listeners, readers and performers experience as the music progresses over time [7].

While music lacks grammatical categories and the syntactic intricacy embedded in linguistic units such as words, it nonetheless exhibits structural organization that parallels language on a more abstract level. Patel [7] (p. 267) refers to this as the syntactic architecture of musical and linguistic sequences, an underlying cognitive framework that governs how elements are hierarchically arranged and processed over time. Both domains appear to engage basic principles of syntactic organization employed by the human mind, such as pattern recognition, prediction, and hierarchical structuring. Given these shared cognitive mechanisms, particularly in how readers of both music and language rely on chunking and syntactic processing to interpret symbolic input, it is meaningful and theoretically grounded to compare music and language reading within a cognitive framework.

The term *chunking* can refer to perceptual organization based on Gestalt principles or musical conventions, while chunking in the working memory sense emphasizes the cognitive benefits of reducing informational load [31]. In music reading, these processes often interact: familiar structures (such as common chord progressions or rhythmic patterns) are more readily chunked, supporting both perception and memory.

Working memory seems to play an important role for efficient sight-reading. Miller’s [32] theory suggested that our working memory can hold about 7 ± 2 chunks of information at once. Cowan’s [33] research suggests that without strategies like rehearsal or chunking based on prior knowledge, the core capacity of working memory is about 4 items. In this study, the term chunk refers to a cognitively meaningful unit—a group of musical elements such as motives, rhythms, or chords that are perceived and processed as a single entity. The concept of chunking is used to describe cognitive mechanisms involved in music reading. In cognitive psychology, deliberate chunking refers to conscious, goal-directed grouping strategies often employed by novices, such as organizing notes by rhythmic or pitch patterns to reduce cognitive load in working memory. Automatic chunking, by contrast, occurs unconsciously in long-term memory and is characteristic of expert performance, where musicians perceive notation as structured, meaningful units, such as melodic phrases or harmonic progressions, rather than isolated symbols [19,31]. Both activities are closely linked to syntactic processing, wherein musical elements are organized hierarchically according to implicit structural rules, much like words and phrases in language. Working memory serves as a temporary workspace where these chunks are activated and manipulated during reading, enabling musicians to integrate incoming visual information with stored musical knowledge [15,34]. Thus, chunking in music reading reflects not only memory strategies but also deeper cognitive processing shaped by experience, musical syntax, and the dynamic interaction between working and long-term memory. In a performance situation, skilled music readers look further ahead in the score, i.e., they have greater eye-hand span than amateur music readers [4,35,36]. This is because they are able to store a larger amount of information in their working memory, compared to less skilled readers. Chunking contributes to reducing cognitive load [32,37].

One consequence of knowing syntactic rules, both in language and music, is the ability to use context to predict that certain elements such as words, chords, or other meaningful units will occur. This ability is crucial for fluent reading of both language and music [15,38,39]. A musical pattern is a sequence of musical elements such as notes, rhythms, chords, or musical structures that creates syntactic order, cohesion, and, therefore, familiarity and predictability in a piece of music. Music reading researchers often use the term *pattern* and *unit* synonymously in this context, describing a succession of several notes [3,14,28]. There are several syntactic strategies related to the grouping of words into meaningful units, segmenting sentences and building new meaningful structures. These syntactic structures also give rise to expectations, allowing readers and listeners to anticipate upcoming material. In music, this predictive processing is supported by tonal and rhythmic regularities, which guide attention and facilitate chunking. Just as readers of language expect certain grammatical constructions to follow one another, skilled music readers anticipate likely continuations based on learned syntactic conventions, enhancing fluency and reducing cognitive load.

As Huron [40] explains in *Sweet Anticipation*, musical expectation is shaped by statistical learning and schematic knowledge, enabling listeners and readers to predict future events based on syntactic cues such as meter, tonality, and phrase structure.

## 3. Eye Movements

When we read, our eyes do not move smoothly scanning the text. We continually make eye movements called *saccades*. Between the saccades, our eyes remain relatively still during *fixations* [1,41]. We do not obtain new information during a saccade, because the eyes are moving too quickly. We process information available within our *perceptual span* during fixations [8]. About 10–15% of the saccades are regressions, going back to previously read words or lines [1].

How long readers look at a word is influenced by how frequent the word is in the language, how familiar it is, and if it is predictable from the preceding context of the sentence. Frequent, familiar and expected words are processed using fewer and shorter fixations than less frequent or unpredictable words, despite the length of the word. Readers tend to skip over high predictable words more frequently than low predictable words [1,8].

Eye movements are also influenced by textual and typographical variables. Fixation duration increases, saccade length decreases, and the frequency of regressions increases while reading a conceptually difficult text. Factors such as the quality of the print, line length, font size and letter spacing influence eye movements, as well [1].

The perceptual span—the effective visual field during reading—has been widely studied using techniques such as the moving window, moving mask, and boundary technique [1]. The moving window technique controls visible text around the fixation point, altering surrounding text to measure how much information is acquired per fixation. The moving mask works inversely, obscuring the fixation area while leaving surrounding text visible. The boundary technique replaces a target word with another until the reader’s eyes cross an invisible boundary, triggering a change; differences in fixation times reveal processing effects.

Like reading language, musicians use saccadic eye movements when reading a sheet of music [42]. Research generally shows that professional music readers exhibit longer saccades and shorter fixation durations compared to less skilled readers [4]. However, fixation behavior is highly variable and influenced by multiple factors, including score complexity, tempo, and familiarity. For example, fixation durations can increase within a single performance depending on tempo or the performer’s growing familiarity with the score [5]. While expert sight-readers tend to show reduced fixation durations, evidence regarding the number of fixations is more mixed, especially when task demands are not controlled. Simpler scores typically lead to fewer and shorter fixations and longer saccades in both expert and novice groups, and repeated exposure to the same score tends to reduce the number of fixations over time [4].

Puurtinen [5] observed that there is a notable limitation of previous eye-movement research in music reading. The main focus seems to place the emphasis on general expertise, which has diverted attention from the effects of the content being read, that is musical stimuli themselves. Madell and Hébert [4] highlighted the need for more research on how stimulus features influence eye movements. Consequently, the research field lacks methodological consistency and has yet to establish a standard approach.

Eye-movement measures are often described as particularly appropriate for studying music reading [4,9]. At the same time, recent meta-analyses summarizing eye-tracking research with focus on differences between expert and non-expert musicians point out contradictory results of the studies describing similar factors [5,10]. These might occur due to not systematically reported age, level of education, and number of years of musical practice for the participants, various features of the experimental set-up, the differences in eye-movement metrics, as well as the chosen stimuli [5,9,10].

Puurtinen [5] in her review of eye movement research focused on four methodological aspects, one of them described as performed music, or ‘what is being read’. The analysis provided highlighted challenges related to variability in the music stimuli and lack of consistency in creating them, especially in studies involving authentic music. Puurtinen [5] concludes:

*Understanding the effects of the most basic features of music notation on the targeting and timing of eye movements seems essential before combining these observations with the effects of expertise, added visual elements, violation of musical expectations in complex settings, or even the distribution of attention between two staves.*

The present study aims to take a closer look at the visual stimuli used in the experiments and examine how their application may influence the results. A detailed analysis of the musical stimuli, considered in light of common music reading strategies across different proficiency levels, may serve as a valuable starting point for more deliberate and informed selection of musical excerpts in future research. A deeper understanding of the nature of musical stimuli can enhance the validity of eye-movement studies by enabling the use of comparable stimuli, facilitating result comparisons, and supporting the replication of experimental conditions.

While music and language differ fundamentally in their relationship to semantic memory (language primarily conveys referential meaning, whereas music does not), the analogy between the two domains remains meaningful at the level of structural organization. Western tonal music, like language, is governed by hierarchical and syntactic principles that shape perception, expectation, and fluency. Musical notation does more than instruct motor actions; it encodes patterns that musicians interpret as coherent auditory structures, organized into phrases and governed by implicit rules. These syntactic properties influence how readers chunk information, predict upcoming events, and process notation efficiently. Therefore, when the research aim is to investigate music reading as a cognitive process, stimuli should reflect these structural conventions rather than consist of random note sequences, just as studies of language reading avoid meaningless letter strings. This perspective does not deny the value of simplified stimuli for studying basic visual-motor mechanisms but emphasizes that higher-level processes require ecologically valid representations of musical syntax.

## 4. Method and Materials

The article presents an analysis of musical stimuli in relation to the core elements that constitute musical syntax essential for fluent reading: melodic, rhythmic, and harmonic patterns [15,26]. Musical stimuli used in eye-movement research typically fall into one of three categories: excerpts from authentic musical pieces, simplified or modified versions of such excerpts, or musical examples composed specifically for research purposes [5,10].

The primary focus of the present study is on musical stimuli that have been composed or arranged explicitly for research. The selection of musical examples aims to illustrate the challenges and potential pitfalls associated with the use of certain stimuli in eye-movement research on music reading over the past few decades. The author does not intend to provide a comprehensive review of recent research in the field, as existing meta-analyses and reviews [4,5,10] already offer an extensive overview of relevant publications.

The publications included in the analysis were selected based on the following criteria: (1) peer-reviewed scientific journal articles, (2) reporting on eye movements during music reading, (3) using musical stimuli specifically composed for research purposes, (4) identifying at least one research aim related to the syntactic processing of musical notation, (5) published in English within the last thirty years (post-1994), and (6) containing figures that depict the musical stimuli used in the study. Criteria related to the mode of stimulus presentation were not applied; studies involving sight reading, rehearsed reading, and silent reading were all considered equally relevant.

Music often involves structural elements such as rhythm, melody, and harmony, though their presence and relative importance vary across genres and instruments [7]. In this study, *melody* refers to the succession of pitch heights perceived as a coherent line. *Rhythm* is understood as the organization of sounds and silences in time, structured into patterns that often convey meter through notation, bar lines, and time signatures. Rhythmic notation typically clarifies the placement of beats and their relative stress, supporting the perception of metric organization [43]. While even, isochronous sequences represent a basic form of rhythm, they provide minimal cues for grouping or phrasing and are therefore not emphasized in this context [44].

Harmony pertains to the relationships among pitches and their connection to the scale or key, indicated by the key signature. It creates contrasts between tension and relaxation. Researchers often describe harmony as groups of simultaneously performed notes [5,26], though this is not an exhaustive representation of the harmonic dimension. A tonal piece consisting solely of a single melodic line has harmonic structure, as the succession of pitches serves to create tension and resolution. Thus, harmony functions as a structural scaffold and a crucial syntactic element in any tonal composition, not only in those featuring multiple simultaneous pitches. This broader understanding of harmony is adopted in the present analysis.

The difficulty of reading and performing the same musical pattern can vary significantly depending on the instrument, due to differences in technique, notation interpretation, and cognitive demands [45]. Musicians tend to focus on aspects of the score most relevant to their instrument. For example, singers often prioritize intervallic relationships and melodic contour, which are essential for intonation and phrasing, while pianists typically attend to harmonic progressions and vertical structures, coordinating multiple voices and hand positions. These findings suggest that instrument-specific strategies play a crucial role in shaping musical expertise and should be considered in both research and pedagogy. These differences were considered in the analysis of the selected studies.

Since several of the analyzed studies use the piano in their experimental design, it may be important to consider the role of fingering in music reading and performance. Pianists must rapidly decide which fingers to use for each note, often in time-limited conditions, and these choices are shaped by motor-anatomical constraints, learned strategies, and musical context. In sight-reading, efficient fingering enables smoother transitions, reduces cognitive load, and supports chunking by allowing the performer to group notes into familiar motor patterns [46]. Ignoring fingering in eye-movement research may overlook a key factor in how pianists process musical stimuli, especially in tasks involving unfamiliar material.

The preliminary analysis included all studies referenced in the reviews by Madell and Hébert [4], Puurtinen [5], and Perra et al. [10]. From this pool, eight publications that met the methodological criteria outlined above were selected for in-depth analysis. Detailed annotations of these studies revealed several overarching themes. Table 1 summarizes the aims, participants, procedures, selected stimuli, and results of the studies.

To illustrate the musical stimuli analyzed in this study, the note sequences were redrawn by the author using Sibelius notation software. These redrawings replicate the original pitch and rhythmic content for clarity. All stimuli are adapted from the cited sources and are presented solely for scholarly analysis. The purpose is to analyze the stimuli used in prior research, similar to quoting sentences used as stimuli when analyzing reading processes.

## 5. Results

The analysis of the syntactic structure of musical stimuli was conducted using key concepts related to the primary elements of musical syntax that facilitate fluent music reading: the use of melodic and rhythmic patterns forming meaningful units, harmonic progression, and the presence of musical motives/phrases or repetitions. An additional aspect considered was the layout of the stimuli, which in some cases did not adhere to conventional rules of musical notation. Given the close relationship between melody and harmony and the fact that only one of the examples involved simultaneously played pitches, the analysis was organized into four categories: (1) melody and harmony, (2) rhythm, (3) phrases and/or repetitions, and (4) layout. Another theme that emerged during the analysis was the researchers’ interpretation of the term “complexity” in relation to musical stimuli.

### 5.1. Study 1 [42] 

**Figure 1:**
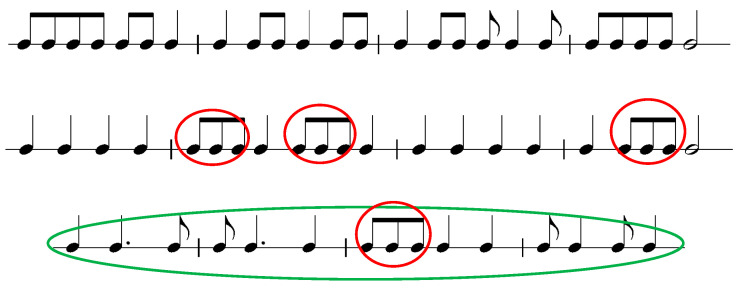
Musical stimuli adapted from Figures 1, 3 and 6 in Kinsler and Carpenter [42]. The notation has been redrawn by the author to illustrate the original rhythmic content. Green and red circles mark rhythm positions that create metrical ambiguity.

Figure 1 presents the musical stimuli used in the study. Red and green markings are added by the author of this paper.

Melody and Harmony: Not Present.

Rhythm: The time signature is not provided, and essential symbols such as triplet markings are missing, making the rhythmic interpretation ambiguous. For example, the beamed eighth notes marked in red could be interpreted as triplets, but the triplet symbol (the number 3 with a bracket) is absent. Similarly, the bars marked in green vary in beat count or assumed meter: bar one suggests 3/4, bar two 3/4 but incorrectly notated, bar three is undefinable (3,5 beats, or 3 beats if the beamed notes are triplets), and bar four could imply 6/8. This ambiguity contrasts with the authors’ own description of the stimuli: “The arrangement and grouping of the notes followed normal musical conventions, with barlines and beams for multiple quavers, except where indicated” [42] (p. 1448). The examples clearly deviate from standard conventions, which increases cognitive load for readers and undermines the assumption of conventional grouping. While such unidiomatic notation does occur in certain repertoires, it typically appears within a defined metric context and assumes performer expertise, conditions absent in these stimuli.

Phrases and/or repetitions: in some cases there might be spotted recurring rhythmic patterns. Layout: only partly following conventional notation.

### 5.2. Study 2 [47]

**Figure 2:**
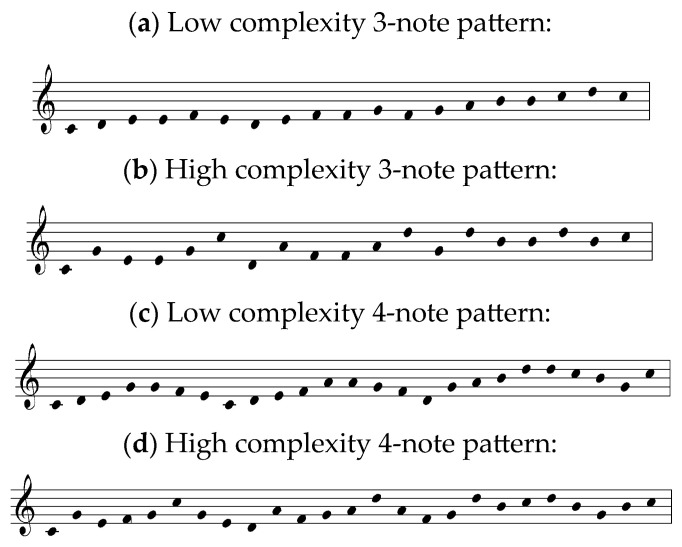
Musical stimuli adapted from Figure 1 in Polanka [47]: low and high complexity patterns consisting of three or four notes. The notation has been redrawn by the author to illustrate the original pitch and rhythmic content.

Figure 2 presents the musical stimuli used in the study.

Melody and harmony: The stimuli are not written in any particular key, although the absence of key signature and the use of C major triads in some of the melodies indicate the key of C major. The researcher describes the stimuli in a following way: “The three-note pattern melodies were designed such that one melody was composed of low complexity patterns (stepwise patterns), one was composed of medium complexity patterns (third-skip patterns and root position triads),and one was composed of high complexity patterns (triads in inversion and broken triads)” [47] (p. 179). The patterns are not visually highlighted in any way, and the conventional notation rules are not used. The stimuli are presented as a succession of dots defining pitch height. This way of writing music seems to be the invention of the researcher, and it does not exist in music literature. Apart from the C major triad, no other harmonic structures can be identified.

Rhythm: Neither the time signature, bar lines nor note values are provided. No patterns can be recognized.

Phrases and/or repetitions: not present. Layout: not following conventional notation.

### 5.3. Study 3 [23], Experiment 2

**Figure 3:**
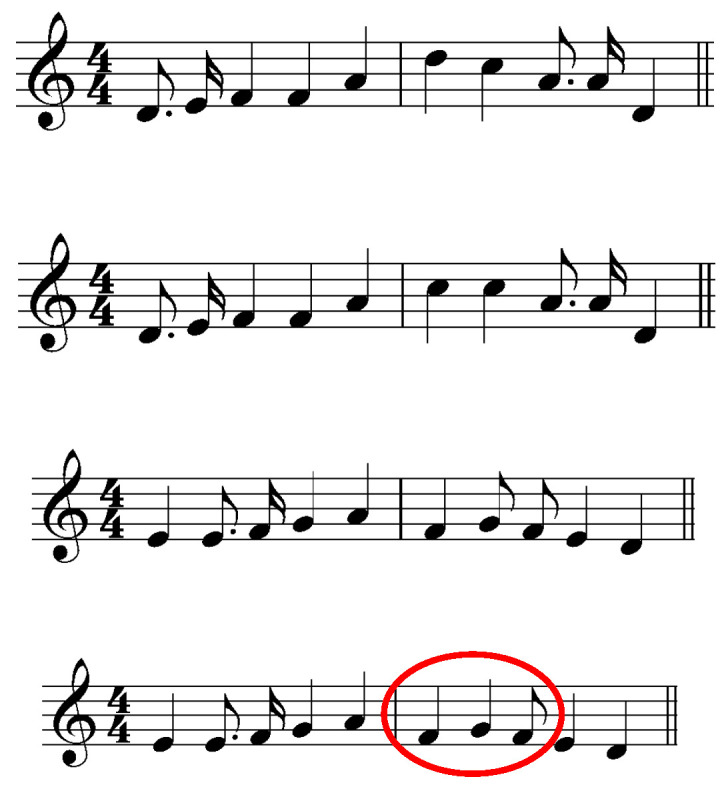
Musical stimuli adapted from Figures 3 and 4 in Waters et al. [23]. The notation has been redrawn by the author to illustrate the original pitch and rhythmic content. Red circle marks rhythm position that create metrical ambiguity.

Figure 3 presents the musical stimuli used in the study. Red marking is added by the author of this paper.

Melody and harmony: the absence of key signature or accidentals suggests the key of C-major in all the examples. However, the pitches seem to be chosen randomly and do not match the key. They do not constitute any syntactically logical melodic or harmonic units of information.

Rhythm: The time signature 4/4 and the bar lines indicate strong and weak beats and the expected groupings of notes. All note values faster than quarter notes are notated using flags. The contemporary rhythm notation practice does not use flags in the way they were used here [44]. The notation of eight and sixteen notes using flags instead of beams is common only in older *vocal* music (never instrumental) and is considered archaic. The bar marked red in Figure 3 violates the syntactic rules in several ways: a half beat is added, and the notation is incorrect.

Phrases and/or repetitions: the pitch and rhythm succession might be considered as musical phrases the way they are presented. Repetitions are not present. Layout: for the most part following the conventional notation

### 5.4. Study 4 [21]

**Figure 4:**
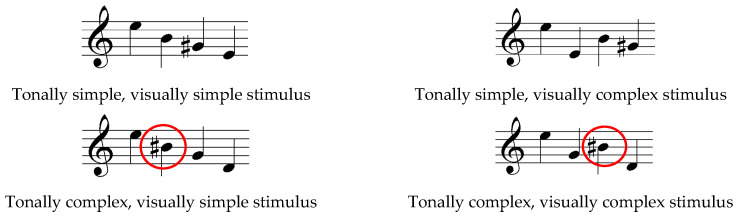
Musical stimuli adapted from Figure 1 in Waters & Underwood [21]. The notation has been redrawn by the author to illustrate the original pitch and rhythmic content. The red marking indicates the use of the note B♯, which is relatively uncommon.

Figure 4 presents the musical stimuli used in the experiment. Red markings are added by the author of this paper.

Melody and harmony: The stimuli consist of an arpeggio of four notes, either tonal (major) or using random pitches, but preserving a visual contour of an arpeggio (no stepwise motion). There is no deliberate use of a melody as an element of the stimuli. Visual complexity is varied by the direction of the subsequent pitches (only ascending/descending or both). Tonal complexity is created by changing the position of accidentals or shifting one or two of the notes to not be encompassed with one diatonic scale. The pitches in “tonally simple” examples constitute a form for harmony—a major chord. It is interesting to note that the researchers chose to use the accidental (#) for the note B (Figure 4, marked red), which is relatively unusual, as B# is enharmonically a C.

Rhythm: Time signature or bar lines are not provided; all the examples consist of four quarter notes. The rhythm is not an element of the stimuli.

Phrases and/or repetitions: not present. Layout: partly following conventional notation.

### 5.5. Study 5 [48]

**Figure 5:**
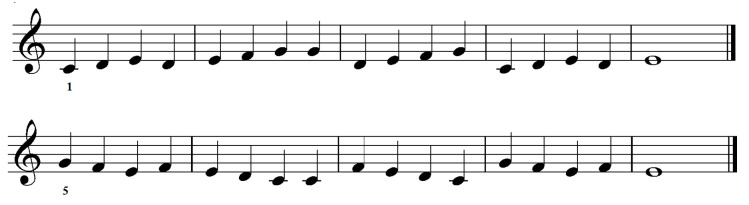
Musical stimuli adapted from Figure 1a,c in Penttinen & Huovinen [48]. The notation has been redrawn by the author to illustrate the original pitch and rhythmic content.

Figure 5 presents musical stimuli used in the study.

Melody and harmony: The stimuli are written in C major, using the first five tones of the scale. The succession of pitches constitutes a form for melody, despite the lack of any harmonic variation. The absence of harmonic progression is the reason why it is not possible to recognize any syntactic units. Provided finger numbers are placed under the note symbols and not above, which is an unusual practice for right hand notes.

Rhythm: No time signature is indicated. The stimuli consist solely of evenly spaced quarter notes arranged in a stepwise pitch sequence. They do not contain rhythmic patterns beyond a basic pulse, as the notes occur at equal intervals without variation in duration or stress (except from the last bar).

The bar lines indicate a 4/4 measure. There are no rhythmic patterns, as the stimuli consist of quarter notes only (except the last bar).

Phrases and/or repetitions: not present. Layout: following conventional notation rules.

### 5.6. Study 6 [49]

**Figure 6:**
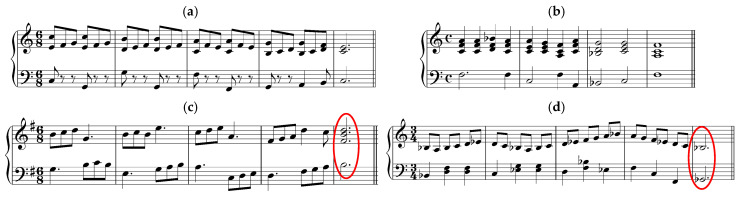
Musical stimuli adapted from Figure 3 in Ahken et al. [49]. The notation has been redrawn by the author to illustrate the original pitch and rhythmic content. Red markings indicate harmonically incongruent chords.

Figure 6 presents the musical stimuli used in the study. Red markings are added by the author of this paper.

Melody and harmony: The examples are written in different keys, typically indicated by a key signature at the beginning of the excerpt. However, in two cases, the key is established solely through accidentals, an uncommon practice that may influence how participants perceive and process the musical material. All the examples consist of syntactic patterns following the musical rules, both melodic and harmonic. The chosen incongruities (marked red in Figure 6) are not random pitches played simultaneously, but harmonically unexpected chords (B minor in example c, and Gb major in example d).

Rhythm: The stimuli follow notational conventions; however, in example d, the eight notes in a 3/4 time signature are beamed in pairs rather than all together, which deviates from standard practice. This atypical beaming may affect how the rhythm is visually processed and interpreted by participants. The stimuli are provided with time signature, bar lines, and the rhythmic patterns are present in all the examples.

Phrases and/or repetitions: there are both melodic and rhythmic motives and repetitions in all the stimuli, used logically, following the common conventions. Layout: following conventional notation rules.

### 5.7. Study 7 [18]

**Figure 7:**
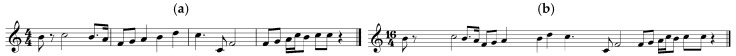
Musical stimuli adapted from Figure 1 in Arthur et al. [18], using normal spacing (**a**) and disrupted spacing (**b**). The notation has been redrawn by the author to illustrate the original pitch and rhythmic content.

Figure 7 presents the musical stimuli used in the study.

Melody and harmony: The lack of key signature suggests the key of C major. However, the succession of pitches seems random and is syntactically incongruent with the key. There is no harmonic progression, and melodic or harmonic patterns are not present.

Rhythm: The examples use a combination of varied note values. Example (a) is supposed to be used as a congruent stimulus, whereas example (b) is manipulated so that “notational structure is unexpectedly changed”. The time signature and the bar lines in example (a) establish the 4/4 m and give expectation of strong and weak beats, as well as note groupings. Notation does not violate the conventions. However, the chosen note values do not constitute any patterns and do not give any logical context for more than one beat at the time. They seem to be randomly put together and are not similar to authentic tonal music pieces.

Phrases and/or repetitions: not present. Layout: following conventional notation rules.

### 5.8. Study 8 [50], Experiment 2

**Figure 8:**
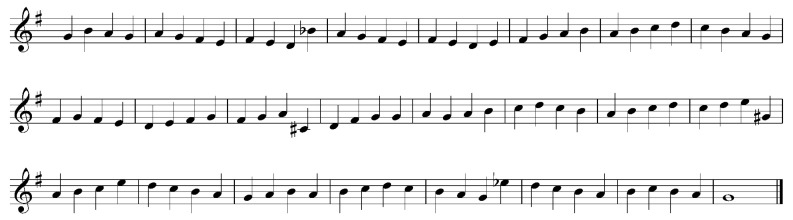
Musical stimuli adapted from Figure 8 in Huovinen et al. [50]. The notation has been redrawn by the author to illustrate the original pitch and rhythmic content.

Figure 8 presents the musical stimuli used in Experiment 2 of the study.

Melody and harmony: The example above is notated in the key of G major; however, the succession of pitches appears largely random and lacks clear patterns or syntactic coherence. While some melodic patterns may evoke expectations of continuation, they tend not to resolve or develop in a way that supports a coherent musical structure. As a result, any performer expectations that arise may be confounded by the lack of harmonic progression.

Rhythm: The example consists solely of evenly spaced quarter notes arranged in a stepwise pitch sequence across 24 bars. No time signature is indicated, and the bar lines only suggest a regular grouping of four beats per measure. Because the notes occur at equal intervals without variation in duration or stress, the stimuli do not contain rhythmic patterns beyond a basic pulse.

Phrases and/or repetitions: not present. Layout: following conventional notation rules.

In all of the analyzed studies, one of the stated aims was to investigate *syntactic processing* in music reading (see Table 1) by examining eye movements. Western tonal music is syntactic, and all authentic (tonal) music scores are composed of melodic, rhythmic, and harmonic patterns that skilled readers instinctively chunk into larger, meaningful units [2,15]. While all of the studies described such elements—musical patterns or structures—in their methodology, the analysis indicates that only a few researchers actually incorporated syntactic patterns into the design of their musical stimuli. Table 2 summarizes the use of the most common syntactic elements across the analyzed studies.

Almost none of the musical stimuli analyzed in the reviewed studies consisted of fundamental units of information essential for structuring single notes into meaningful units—an ability crucial for efficient sight-reading, defined here as the unrehearsed performance of notated music. Nevertheless, sight-reading was one of the primary data collection methods, employed in five of the eight experiments.

In contrast, experiments investigating eye movements during language reading often focus on the reader’s comprehension when various textual elements are manipulated [8]. Reading a sentence out of context typically does not hinder understanding, as the syntactic structure remains intact. Similarly, reading a sequence of notes following the syntactic rules and constituting a melody allows understanding. However, reading a sequence of music notes that lacks harmonic, melodic, or rhythmic coherence fails to provide meaningful connotations for a musician, defined as logical and expected successions of events governed by syntactic rules [15].

### 5.9. Complexity of Musical Stimuli

In several of the reviewed publications, the term *complexity* is frequently used. However, the level of complexity in sheet music appears to be a subjective concept that is difficult to define and verify.

Polanka [47] conducted an experiment using musical examples with varying levels of complexity. Scalar patterns were classified as low-complexity, root position triadic patterns as medium-complexity, and inverted triadic patterns as high-complexity (Figure 2). Waters and Underwood [21] also investigated how musical complexity influences eye movements, using short monophonic examples consisting of four notes (Figure 4). The researchers described their stimuli as follows (p. 49):

*Twenty “Tonally Simple, Visually Simple” stimuli were composed with four notes preceded by a treble clef, forming simple scale or arpeggio structures. All notes fit within a single major diatonic scale, each containing two or fewer accidentals and one or no contour changes. Twenty “Tonally Simple, Visually Complex” stimuli retained the same musical structures but were arranged to include two contour changes. Twenty “Tonally Complex, Visually Simple” stimuli were created by altering one or two notes so they no longer fit within a single diatonic scale, or by repositioning accidentals. Finally, twenty “Tonally Complex, Visually Complex” stimuli combined these tonal alterations with two contour changes.*

This description illustrates how both visual and tonal complexity in a four-note pattern can be manipulated by modifying individual notes or accidentals. Kinsler and Carpenter [42] approached complexity through rhythmic patterns, defining complex stimuli as those that violate conventional music notation rules (see Figure 1).

Huovinen and colleagues [50] also addressed the concept of complexity, interpreting it as expected processing load. They proposed that larger melodic intervals pose greater cognitive challenges than smaller, stepwise ones, which may be more easily decoded as directional commands (up/down) within a scale. Accordingly, stimuli featuring larger intervals or accidentals can be considered more complex than those using stepwise motion and diatonic pitches.

### 5.10. Unexpected Findings

An analysis of the results and discussions in the reviewed publications revealed that several findings were described by the researchers as difficult to explain or unexpected.

Kinsler and Carpenter [42] (p. 1450) observed:

*Sometimes a subject may fixate each of a pair of quavers individually, sometimes only one of them, or neither. (…) Comparison of the number of saccades made when performing musically identical bars with quavers notated either as isolated or beamed showed, for two subjects, a significant (p = 0.05) decrease with beaming, but for the other subject (RHSC), an equally significant increase—demonstrating again the idiosyncratic nature of the responses.*

Polanka [47] (p. 182) noted: “It had been assumed that stepwise patterns were the least complex musically and therefore would be processed in the largest units. This was not found to be the case.” Waters et al. [23] found that musician groups made more errors on the duration-different trials than nonmusicians, and vice versa for the pitch-difference trials. The authors explained this as follows (p. 486): “The violation of the space-duration relationship in generating duration differences probably results in the tendency of the musician subjects to overlook the duration ‘misprints’.” Waters and Underwood [21] (p. 58) also reported an unexpected result:

*However, on the first stimulus presentation, it is interesting that there was no evidence that the tonal complexity of the material had any effect on the fixation durations of the experts, as might be predicted from Kinsler and Carpenter’s model. This is a curious finding since we would have expected the experts’ drop in accuracy on the more difficult material to have been due to encoding difficulties on the first stimulus presentation. Furthermore, there was no suggestion from the spatial data that the experts used larger saccade sizes for the tonally simple material. In other words, there was no evidence for any differences in eye movement behaviour between tonally simple and tonally complex material for the expert group.*

Arthur et al. [18] similarly reported: “Score disruption had no significant effect on the Total Time within either group. Saccadic latency was the only other measure to reach significance, and this was for experts only when encountering disrupted score—the latency increased significantly”.

Interpretation of these findings in the context of syntactic processing will follow in the next section.

## 6. Discussion

The syntactic properties of written music appear to have been insufficiently considered in the design of musical stimuli in most of the analyzed studies. The following section discusses the degree to which these stimuli align with the syntactic conventions of Western tonal music.

### 6.1. Melodic and Harmonic Patterns

Knowledge of the key in which a piece or excerpt is written provides a set of expectations for an experienced sight-reader. For example, an authentic four-bar melody typically follows a harmonic progression that alternates between tension and resolution. Notes on strong beats are expected to be chord tones, complemented by passing tones on weak beats. Musicians also develop motor expectations, particularly pianists, who often play multiple tones simultaneously. A key signature with one sharp (♯) will prompt a pianist or violinist to position their hands according to the G major triad and anticipate D major tones in subsequent bars.

Of the studies analyzed, only one—by Ahken et al. [49], in which the stimuli were composed by a graduate student in music composition—explicitly incorporated harmonic progressions in the musical stimuli. While several other studies declared a key and included a key signature, this often amounted to using seven notes from a diatonic scale without further harmonic development [48,50]. For example, Huovinen et al. [50] include some basic harmonic structure, such as beginning and ending on the tonic and using familiar melodic cells, which may evoke tonal expectations. However, these features are limited and do not constitute full harmonic progressions, making their role in supporting syntactic processing ambiguous.

Some studies did not specify a key at all, yet still examined how participants organized notes into patterns [23,47], or stated that the stimuli used “white notes only” to investigate visual expectations in sight-reading [18]. As a result, although some stimuli may visually resemble Western tonal melodies, their melodic and harmonic content often fails to follow fundamental syntactic rules. Consequently, common melodic or harmonic patterns are absent. Even when using the same pitches and intervals, a slight reordering, if aligned with syntactic rules, can significantly alter visual processing. Stepwise motion and intervallic skips become meaningful only when they can be chunked as part of a scale or harmonic progression expected in a given key.

A skilled sight-reader typically assumes that the absence of a key signature indicates C major (or A minor). If the tonal material contradicts this assumption (see, e.g., Figure 3 and Figure 6), it may disrupt cognitive processing due to perceived incongruity. This is analogous to how a sequence of letters is chunked into words based on syntactic meaning, not merely visual similarity.

### 6.2. Rhythm Patterns

Note values were an active component of the stimuli in four of the studies: Kinsler and Carpenter [42], Waters et al. [23], Arthur et al. [18], and Ahken et al. [49]. Of these, only the stimuli used by Ahken et al. [1] consisted of common rhythmic patterns that could plausibly appear in authentic musical compositions. The other three studies either violated conventional rules of music notation or used a variety of note values placed relatively randomly within each bar. None of these stimuli resembled authentic rhythmic patterns. Moreover, some studies did not include a time signature at all [47,48,50]. Yet the time signature is an essential element in rhythm reading, as rhythmic units can only be interpreted meaningfully in relation to meter and beat.

The analysis of rhythmic content in the reviewed studies reveals that most researchers did not employ familiar rhythmic patterns or incorporate any form of repetition in the succession of rhythmic events. As a result, the syntactic processing of these musical stimuli may differ substantially from that involved in reading authentic rhythmic sequences.

Several stimuli lacked differentiated rhythmic information altogether. In some cases, they consisted only of dots indicating pitch [47] or used the same note value throughout the entire excerpt [21,48]. This design choice was presumably made to isolate pitch reading and minimize the influence of other musical elements. However, a melody line without varied note values, and in one case, even without bar lines (see Figure 2), is visually unnatural and rare in authentic musical scores. Beyond their role in conveying meter and accentuation, bar lines also serve a referential and visual function [48].

Chunking information into larger units appears to be particularly relevant to rhythmic processing [45,51]. A useful analogy can be drawn from language: reading sentences composed entirely of capital letters significantly slows processing compared to sentences that follow standard capitalization rules [52]. Similarly, reading pitches without rhythmic differentiation may reduce the efficiency of visual processing in music reading.

### 6.3. Complexity

The term complexity has been interpreted in diverse ways across the reviewed studies, often without a shared conceptual framework. The dimensions of complexity in music notation, both visual, tonal and structural, are often deeply connected to each other. For example, increasing the number of accidentals not only adds to the visual density of the score but also introduces greater tonal ambiguity. Similarly, scalar and triadic patterns may be considered both as tonal structures and as contributors to visual or structural complexity, depending on the context. Waters and Underwood [21] classified accidentals as a visual element of the score rather than a tonal one, manipulating visual complexity by increasing the number of accidentals. In contrast, Huovinen et al. [50] treated the use of accidentals as a form of cognitive complexity. Polanka [47] considered scalar and triadic patterns as components of tonal complexity, as opposed to visual complexity. These divergent interpretations highlight the need for a more systematic approach to how complexity is conceptualized and operationalized in music notation research. Several aspects could be investigated in this context:

- Does the use of accidentals contribute to tonal complexity, visual complexity, or both? In a musical context, accidentals increase harmonic complexity, but they also make the score visually more complex.
- Do scales and triads affect tonal or visual complexity? These are basic and high-frequency musical structures.
- Can we meaningfully discuss musical structure, tonal progression, and complexity when the stimulus consists of only four notes, as in Waters and Underwood [21]?

This inherent overlap makes it difficult to draw strict boundaries between categories of complexity. Rather than treating these dimensions as mutually exclusive, it may be more productive to view them as interrelated aspects that together shape the overall processing demands of a musical score. Future research should therefore clearly articulate which aspects of complexity are being manipulated and acknowledge the multidimensional nature of these constructs.

Given the multidimensional nature of musical notation, it may also be beneficial to conceptualize complexity as a spectrum or network of interacting features, rather than as discrete categories. For instance, accidentals can be coded as both visual and tonal features, and their impact may depend on the specific research question or experimental context. Researchers might consider using a matrix or coding scheme that allows elements to contribute to multiple dimensions of complexity simultaneously.

Another possibility is to define categories of complexity, distinguishing between notational, cognitive, and performance complexity. This approach recognizes that a single notational feature may simultaneously increase visual load, cognitive processing demands, and performance difficulty, depending on the context and the performer’s background:

- Notational complexity: all graphical features, including density, clefs, accidentals, rhythmic patterns.
- Cognitive complexity: the mental effort required to process the notation, including tonal ambiguity, unfamiliar patterns, or non-standard structures.
- Performance complexity: the demands placed on the performer, including motor planning, fingering, and instrument-specific challenges.

Eye-tracking measures such as fixation duration and saccade length are commonly used to assess processing demands. They could be treated as indicators of processing difficulty, and in this way contribute to defining complexity. These metrics may reflect cognitive load, but they are influenced by multiple factors and do not offer a direct or exclusive measure of complexity. A more productive approach would be to examine how different operationalizations of complexity correlate with behavioral and physiological indicators, allowing for a more nuanced understanding of how complexity affects music reading and performance.

In the absence of a standardized framework, researchers should clearly articulate the rationale behind their categorization of musical elements and specify which aspects of complexity are being manipulated. This would enhance comparability across studies and support the development of a more coherent conceptual model for musical stimulus complexity.

### 6.4. Music Instrument and Motor Planning

The difficulty of reading and performing the same musical pattern can vary significantly depending on the instrument. It can also be assumed that musicians playing different instruments focus on different aspects of the score to optimize performance. For example, singers may naturally focus on intervals, while pianists are more likely to attend to harmonic progressions. However, none of the reviewed studies discussed their results in light of instrument choice (most often the piano) or considered the broader motor aspects that may influence eye movements during sight-reading.

Two of the studies [48,50] investigated the processing of intervals using the piano. The authors compared visual processing of stepwise motion with that of intervallic skips. The piano is one of the few instruments where the spatial distance between notes on the staff corresponds directly to the number of keys skipped on the instrument. For example, the interval of a fifth (C–G) can be challenging for novice pianists, both due to the physical span and the use of the fifth finger (pinky), which is often less developed in beginners. In contrast, the same interval poses no motor challenge for a novice trumpet player, who can play both notes without changing finger positions—unlike stepwise motion, which may require more complex motor planning. Beginning brass players may not rely on scale-based visual strategies in the same way pianists do, due to the differing properties of their instruments.

Another example is the stepwise interval C5–D5, which is relatively easy to play on many instruments but can be challenging for beginner flutists due to the motor complexity involved. Huovinen et al. [50] define complexity as expected processing load and suggest that larger melodic intervals involve greater cognitive difficulty than smaller, stepwise ones. While this may hold true for pianists, excluding the motor characteristics of the instrument in more general discussions of visual processing during sight-reading may compromise the validity of the findings.

An additional factor related to piano performance is fingering. Fingering is an integral part of piano notation and may be crucial for successful sight-reading [46]. Two of the analyzed studies [48,50] included finger numbers at the beginning of five-finger stimuli played by novices. However, the same authors did not include fingering in their most complex musical stimuli (see Figure 8). Similarly, fingering was not provided in the stimuli used by Ahken et al. [49]. This suggests that the researchers may not have fully considered the physiological demands of piano playing when designing their stimuli.

For instance, the stimulus shown in Figure 8 could likely be sight-read with relative ease by a trumpeter but would be more challenging for a (non-expert) pianist, who must plan awkward fingerings in real time. It is possible that presenting the same notes in a different order would have yielded different results. Some of the musical stimuli that were presumably considered easy to play by the researchers may, in fact, be quite demanding—precisely because they do not account for the motor aspects of performance.

### 6.5. Interpretation of the Unexpected Findings

Some of the results reported in the reviewed studies appeared difficult for the researchers to explain. Kinsler and Carpenter [42] discussed why participants sight-read the same stimuli in contrasting ways. These differences in processing may be attributed to the syntactic context and the presence or absence of a priming effect (see Figure 1) [39]. Although no participant data were provided, it is reasonable to assume that novice readers may have lacked experience with reading eight notes notated with flags instead of beams—a style rarely used in instrumental music. Conversely, such atypical notation might have been perceived as incongruent by experienced musicians, while novices may not have recognized it as unusual.

Polanka [47] reported that stepwise patterns were not processed in an expected way: in larger units, compared to patterns involving larger intervals. A closer examination of the stimuli (Figure 2) suggests that the visual layout of the stepwise motion may have introduced challenges not typically encountered in conventionally notated music, where bar lines and rhythmic differentiation facilitate chunking. Although the researcher used 3- and 4-note patterns, there were no visual cues in the stimuli to support the reader in recognizing these groupings.

Waters et al. [23] found that musician groups made more errors on duration-different trials than nonmusicians, and vice versa for pitch-difference trials. The authors attributed this to musicians overlooking the duration “misprints.” An alternative explanation may lie in the violation of notational conventions: the extensive incongruities in the duration-different trials may have disrupted chunking for musicians, whereas nonmusicians—lacking such expectations—were unaffected. Altering a single pitch in a bar is a relatively minor change, but adding half a beat (as in Figure 3) disrupts the entire metrical structure.

Waters and Underwood [21] reported no significant differences in eye movement behavior between tonally simple and tonally complex material among expert participants. This may be due to the shortness of the stimuli—only four single notes (see Figure 4). Such short excerpts, lacking established tonality, may prompt a note-by-note reading strategy regardless of expertise. Chunking typically requires harmonic, melodic, or rhythmic context. Moreover, chunking enables the simultaneous processing of approximately four (plus or minus two) elements in working memory [33]. A task involving only four elements can be completed accurately without chunking or relying on strategies unique to expert readers.

Arthur et al. [18] found no effect of score disruption on total task time for either expert or nonexpert participants. Figure 6 shows that the “non-disrupted” musical stimulus lacked any common melodic, rhythmic, or harmonic patterns. It is therefore plausible that expert readers processed the stimulus similarly to nonexperts—a phenomenon comparable to findings in chess research, where experts perform no better than novices when presented with random, non-meaningful configurations [53].

### 6.6. Comparison to Linguistic Stimuli

Reading a succession of pitches without any musical context—such as key signature, harmony, or phrasing—likely provides a level of understanding comparable to reading a series of unrelated words or non-words. This type of visual stimulus removes much of the essential information that skilled music readers rely on to decode a score efficiently, as they would in everyday performance situations. As a result, such stimuli may significantly affect research outcomes, particularly in studies comparing novice and expert readers, since beginners do not utilize contextual musical knowledge to the same extent as experienced musicians [2,28].

The process of reading individual, randomly placed letters differs fundamentally from reading words or meaningful groups of words. In language reading research, eye movement studies often use visual stimuli such as words and syntactically coherent sentence elements, at least as a part of the stimulus. They rarely use single letters or solely non-words. It is unclear why some music reading studies treat single notes as meaningful units, rather than focusing on melodic, rhythmic, or harmonic patterns.

The musical stimuli used in the analyzed studies vary widely in their degree of syntactic structure and potential for chunking. Some, like those in Kinsler & Carpenter [42] and Polanka [47], rely on abstract or visually unconventional representations—such as rhythmic patterns without time signatures or pitch dots spaced evenly on a staff—making them comparable to succession of unrelated or distorted words in language reading. Others, like Waters et al. [23], present short excerpts with intentional violations of tonal and rhythmic norms, resembling non-word sentences that lack grammatical and semantic coherence. In contrast, Ahken et al. [49] incorporate harmonic and rhythmic patterns within a clear key and meter, offering a more structured musical context, similar to meaningful sentences with a syntactic or semantic incongruity at the end. These differences underline the importance of considering syntactic coherence when interpreting findings in music reading research, as the potential for chunking and expectation formation varies significantly across stimulus types.

While the reviewed studies exhibit notable limitations in the design of musical stimuli, particularly in their treatment of syntactic elements such as melodic, harmonic, and rhythmic patterns, they nonetheless represent important contributions to the emerging field of eye movement research in music reading. These studies have laid foundational groundwork by demonstrating the feasibility of applying cognitive science methodologies to musical contexts and by highlighting the influence of visual features on music reading. Their varied approaches to complexity, though inconsistently defined, have opened valuable discussions about how musical notation is perceived and processed. Moreover, by attempting to isolate specific variables such as pitch or visual layout, these studies have provided insights into the perceptual strategies employed by musicians, especially in relation to expertise. The methodological gaps identified here offer a roadmap for future research, underscoring the need for syntactically coherent stimuli and standardized conceptual frameworks. In this way, the studies not only inform current understanding but also catalyze the refinement of experimental design in music cognition research.

## 7. Conclusions

This study highlights a critical gap in the design of musical stimuli used in eye-movement research on music reading. While linguistic reading research often employs syntactically meaningful units such as words and sentences, many music reading studies rely on stimuli that lack equivalent syntactic coherence—melodic, rhythmic, and harmonic patterns essential for fluent music reading. The analysis revealed that only one of the eight reviewed studies incorporated musical stimuli that could realistically be part of an authentic tonal composition.

The absence of syntactic structure in most stimuli compromises the validity of findings, particularly when comparing syntactic processing in expert and novice readers. Furthermore, inconsistent interpretations of complexity and a lack of consideration for motor planning and instrument-specific challenges further obscure results. To advance the field, future research must adopt standardized definitions of musical complexity, ensure stimuli reflecting authentic syntactic structures, and account for the motor demands of different instruments. Doing so will enhance the reliability and comparability of findings and support the development of a robust framework for understanding visual processing in music reading.

## Figures and Tables

**Table 1:** Overview of eight empirical studies on eye movements in music reading, including research aims related to syntactic processing, participant characteristics, types of musical stimuli, experimental procedures, and key findings.

Study	Aim (Related to Syntactic Processing)	Participants	Stimuli	Procedure	Conclusion
(1) Kinsler & Carpenter, 1995 [42]	To examine saccades while reading music: to be able to dissociate the contributions of factors associated with the input (the printed music) and with the output (the rate of execution).	No data.	Short rhythmic phrases presented as a single line of notes with bar lines.	Participants were suddenly presented with a line of notes on a computer screen and asked to tap the corresponding rhythm on a microphone.	At slow speeds with complex sequences there may be considerably more saccades than notes; with a fast speed and simple pattern—shown here as more notes than eye movements.
(2) Polanka, 1995 [47]	To determine whether musicians read in higher order structures (patterns) or notes by monitoring their eye movements as they sight-read.	18 undergraduate music majors (11 females, 7 males), divided into three skill groups based on a sight-singing pretest.	Six melodies—three composed of three-note pitch patterns and three of four-note patterns, each with varying complexity (low, medium, high).	Subjects read each melody twice, once silently and once humming and their vocal responses were recorded on audio tape.	Better readers tended to process larger units than poorer readers. Pattern size influenced eye movement behavior. Stepwise patterns were processed in smaller units than triadic patterns.
(3) Waters et al., 1997 [23] exp.2	To test whether skilled sight-reading is associated with rapid processing of note groups and to examine the relationship between expertise and eye-movement parameters (e.g., fixation duration).	Three groups of 8 subjects each: two “expert” groups (full time music students playing a monophonic instrument) and a novice group (familiar with the names of the notes).	Sixty 10-note melodies in 3/4 or 4/4 time, each consisting of two bars of five notes each. No key signature or accidentals. Each melody had a randomized counterpart.	Silent reading, matching pairs of stimuli as same or different by pressing a button as quickly as possible.	Experienced musicians used larger units and processed them with fewer and shorter fixations. Musicians made more errors on duration-different trials, while nonmusicians made more errors on pitch-different trials.
(4) Waters & Underwood, 1998 [21]	To determine the effect of the tonal complexity of the stimuli on task performance and eye movement behaviour. To determine whether there was any difference in task performance and eye movement behaviour for expert and novice musicians.	Twenty-two subjects divided into two groups: “expert” group, experienced musicians playing at least one musical instrument associated with the treble clef register. The “novice” group, familiar with musical notation.	Twenty “Tonally Simple, Visually Simple” stimuli: four notes encompassed within one major diatonic scale, preceded by the treble clef, consisting of simple scale or arpeggio structures. Other stimuli with various complexity level were created by shifting some of the notes.	Silent reading. Each subject made a “same” response with their preferred hand, and a “different” response with their non-preferred hand.	Experts outperformed novices in speed and accuracy. Experts showed reduced performance on tonally complex material, while novices showed no difference. There was no evidence for any differences in eye movement behavior between tonally simple and tonally complex material for the expert group.
(5) Penttinen & Huovinen, 2011 [48]	To elucidate the early stages of learning to read music in adulthood by examining the various measures of fixation time in elementary sight-reading tasks and comparing novices with experienced music amateurs.	49 s-year teacher education students in Finland, all enrolled in a year-long compulsory music course.	Twelve five-bar melodies in C major, using quarter notes and a whole note in the final bar. Melodic range: C4–G4. Fingering marked for the first note. The melodic movement in each melody was primarily stepwise, with the exceptions of two larger intervals at the temporal distance from one another.	Participants sight-read four melodies on piano with a metronome (60 bpm) at three time points: start, mid-point (16 weeks), and end of the course.	Sight-reading skills improved significantly. Fixation times decreased for central notes in large intervals, but not for surrounding notes.
(6) Ahken et al., 2012 [49]	Investigating the eye movements of readers during the visual processing of music and linguistic syntactic incongruities. To examine the role of key signature and accidentals to establish tonality.	Eighteen experienced pianists.	Sixteen short musical phrases (5–7 bars), grouped in fours. Half were syntactically congruent; the rest ended with a non-tonic chord or note.	Participants were instructed to play each musical sequence at the piano with hands together and no preview time, at any speed they liked.	Incongruent stimuli elicited more fixations, longer fixation durations, and longer trial durations. Effects were less pronounced for stimuli with accidentals than for those with key signatures.
(7) Arthur et al., 2016 [18]	To explore how visual expectations influence sight-reading expertise, focusing on working memory, cross-modal integration, and visual crowding. The study examined eye movement responses to unexpected changes in notation observed in expert and non-expert music sightreaders.	9 participants were assigned to the expert sight-reader group and 13 to the non-expert sightreader group. No data about their main instrument.	Ten four-bar melodies in treble clef, right-hand only, using white notes. Notational features were altered (e.g., bar line removal, stem direction, spacing).	Participants sight-read the 9 specifically composed musical excerpts of 4 bars duration on the piano.	Score disruption had no effect on total task time. Saccadic latency increased significantly for experts only when encountering disrupted notation.
(8) Huovinen et al., 2018 [50], exp 2	To examine the hypothesis stating that local increases in music-structural complexity (and thus visual salience) of the score may bring about local, stimulus-driven lengthening of the ETS [eye-time span].	14 professional piano students from three Finnish universities.	Eight mostly stepwise melodies in 4/4 time, each six bars long and composed entirely of quarter notes. The melodies were divided into two sets in the keys of G, C, F, and B♭. In each melody, one larger intervallic skip (a minor sixth) was inserted in one of bars 3–5.	The participants were instructed to sight-read the melodies on the piano in time with a metronome.	Experienced musicians appeared to react sensitively to upcoming deviant elements. Target notes triggered longer-than-average eye-time spans for notes occurring several beats before the target itself. Sight-readers often responded to the target element as early as six beats in advance.

**Table 2:** The use of syntactic elements in musical stimuli of the analyzed studies.

Study	Syntactic Units of Information Possible to Chunk				Could Be a Part of an Authentic Piece
	Melodic	Rhythmic	Harmonic	Phrases/Repetitions	
1 [42]	Not applicable	limited	Not applicable	limited	no
2 [47]	limited	no	no	no	no
3 [23] exp 2	no	no	no	limited	no
4 [21]	no	no	limited	no	limited
5 [48]	no	no	no	no	no
6 [49]	yes	yes	yes	yes	yes
7 [18]	no	no	no	no	no
8 [50] exp 2	no	no	no	no	no

## Data Availability

Data for this study consist of musical examples published in eight open-access articles. These materials can be accessed freely via the original publications listed in the References section.
